# Research Progress on Porcine Reproductive and Respiratory Syndrome Virus

**DOI:** 10.3390/v18010035

**Published:** 2025-12-24

**Authors:** Xingyang Cui, Suzhen Liu, Tongqing An

**Affiliations:** 1 College of Animal Science, Wenzhou Vocational College of Science and Technology, Wenzhou 325006, China; 2 State Key Laboratory of Animal Disease Control and Prevention, Harbin Veterinary Research Institute, Chinese Academy of Agricultural Sciences, Harbin 150069, China

Porcine reproductive and respiratory syndrome (PRRS) is one of the most significant diseases affecting the global swine industry, characterized by reproductive failure and late-term abortion in sows and respiratory diseases in all age of pigs [1,2,3]. At present, PRRS has been reported in most pig breeding countries and regions in the world [3,4]. Despite decades of dedicated research and the implementation of extensive biosecurity and vaccination protocols, PRRS virus (PRRSV) continues to pose challenges to pig farmers due to its remarkable genetic diversity, macrophage tropism, persistent infection, environmental persistence, and ability to evade the host’s immune response.

This Special Issue highlights the ongoing role of recombination in shaping PRRSV genetic diversity and emerging of PRRSV variants. In an epidemiological survey of PRRSV, spanning most provinces of China in 2021–2022, the results revealed that L1 PRRSV (including NADC30-like PRRSV, NADC34-like PRRSV) is the predominant lineage in wild-type PRRSV in China, with a proportion of 32.1% in PRRSV-positive samples [5]. In 2024, Li et al. reports the identification of a novel recombinant PRRSV strain (XJ-Z5 strain) in China. The XJ-Z5 strain is a NADC30-like recombinant virus derived from multiple parental strains, and it is moderate virulent to piglets, with high viral loads in the lungs and persistent shedding [6]. Herrera da Silva and colleagues found an increased recombination frequency by utilizing whole-genome sequencing and recombination analysis. The results suggest that recombination likely drove the emergence of PRRSV L1H.18 clade, illustrating how genetic exchange contributes to novel variant formation in high-density swine production areas [7]. Taken together, these articles emphasize recombination as a key mechanism in PRRSV evolution, and recombinant PRRSV variant show different pathogenicity to piglets.

The recombination frequency is reported to be related with viral fidelity. Fidelity analysis reveals striking differences between a HP-PRRSV (JXwn06) and NADC30-like strain (CHsx1401), CHsx1401 demonstrates a higher recombination frequency, while JXwn06 accumulates more mutations. Importantly, they identify the nsp9-nsp10 region as a critical factor influencing fidelity differences [8]. As elaborated in the review by Cui et al., PRRSV recombination exhibits dynamic patterns, with shifting hotspots across inter- and intra-lineage events [9]. Recombinant strains often exhibit enhanced virulence and replication adaptability compared to parental strains, accelerating the outbreak of the epidemic and economic losses. A critical concern involves recombination associated with modified-live virus (MLV) vaccines, which triggers virulence reversion and undermines vaccine safety. High densities and improper immunization further facilitate viral co-infection and recombination. Strengthening genome monitoring to track emerging variants, rationalizing the use of MLV vaccines, and advancing research on recombination mechanisms may address this issue.

Two additional studies in this issue provide novel perspectives, addressing environmental transmission routes and a promising antiviral intervention. Alvarez-Norambuena et al.’s soil percolation study reveals a previously unrecognized transmission route, all three tested PRRSV-2 strains can penetrate 13 distinct Minnesota soil types, with viral titers decreasing as soil volume increased [10]. This finding links potential groundwater contamination to fall crop harvesting and manure application, activities whose timing coincides with seasonal PRRS outbreaks. It underscores the need for enhanced biosecurity measures in waste management. Concurrently, Si et al. identify SR717, a STING agonist, as a potent anti-PRRSV agent [11]. The compound inhibits multiple PRRSV strains in both Marc-145 cells and PAMs via a dose-dependent mechanism, targeting viral replication, assembly, and release without affecting adsorption or entry. By activating the STING-TBK1-IRF3 pathway, SR717 boosts antiviral cytokines like IFN-β and IL-6, offering a novel therapeutic strategy that may circumvent limitations inherent to current vaccination approaches.

This Special Issue captures the cutting-edge advances in PRRSV research, reflecting significant strides in understanding viral genetics, epidemiology, environmental persistence, and host–pathogen interactions. The collective findings underscore the complex and adaptive nature of PRRSV, propelled largely by recombination and influenced by production practices and ecological factors. Moving forward, advanced genomic surveillance, rational vaccine design, environmental risk assessment, and innovative immunotherapeutic discovery will be essential for developing durable, sustainable control strategies. By synergizing these research domains, the scientific community can better anticipate viral evolution, alleviate the economic and animal welfare burdens of PRRS, and contribute to the stability and security of the global food supply chain.

## References

[1] Albina E. (1997). Epidemiology of porcine reproductive and respiratory syndrome (PRRS): An overview. Vet. Microbiol..

[2] Pejsak Z., Stadejek T., Markowska-Daniel I. (1997). Clinical signs and economic losses caused by porcine reproductive and respiratory syndrome virus in a large breeding farm. Vet. Microbiol..

[3] Neumann E.J., Kliebenstein J.B., Johnson C.D., Mabry J.W., Bush E.J., Seitzinger A.H., Green A.L., Zimmerman J.J. (2005). Assessment of the economic impact of porcine reproductive and respiratory syndrome on swine production in the United States. J. Am. Vet. Med. Assoc..

[4] Xie J., Cui T., Cui J., Chen Y., Zhang M., Zhou P., Deng S., Su S., Zhang G. (2014). Epidemiological and evolutionary characteristics of the PRRSV in Southern China from 2010 to 2013. Microb. Pathog..

[5] Li C., Fan A., Liu Z., Wang G., Zhou L., Zhang H., Huang L., Zhang J., Zhang Z., Zhang Y. (2024). Prevalence, Time of Infection, and Diversity of Porcine Reproductive and Respiratory Syndrome Virus in China. Viruses.

[6] Li H., Zhang W., Qiao Y., Wang W., Zhang W., Wang Y., Yi J., Zhang H., Ma Z., Chen C. (2025). Genome and Pathogenicity Analysis of an NADC30-like PRRSV Strain in China’s Xinjiang Province. Viruses.

[7] Herrera da Silva J.P., Rossow S., Paploski I.A.D., Kikuti M., Corzo C.A., VanderWaal K. (2025). Complete Genome and Recombination Analysis of a Novel Porcine Reproductive and Respiratory Syndrome Virus 2 (Variant 1H.18) Identified in the Midwestern USA. Viruses.

[8] Gao X., Bian T., Gao P., Ge X., Zhang Y., Han J., Guo X., Zhou L., Yang H. (2024). Fidelity Characterization of Highly Pathogenic Porcine Reproductive and Respiratory Syndrome Virus and NADC30-like Strain. Viruses.

[9] Cui X.Y., Xia D.S., Luo L.Z., An T.Q. (2024). Recombination of Porcine Reproductive and Respiratory Syndrome Virus: Features, Possible Mechanisms, and Future Directions. Viruses.

[10] Alvarez-Norambuena J., Quinonez-Munoz A., Corzo C.A., Goyal S.M. (2025). Comparative Adsorption of Porcine Reproductive and Respiratory Syndrome Virus Strains to Minnesota Soils. Viruses.

[11] Si X., Wang X., Wu H., Yan Z., You L., Liu G., Cai M., Zhang A., Liang J., Yang G. (2024). Inhibition Effect of STING Agonist SR717 on PRRSV Replication. Viruses.

